# *catena*-Poly[[(pyrimidine-2-carb­oxy­lic acid)iron(II)]-μ-oxalato]

**DOI:** 10.1107/S1600536810030023

**Published:** 2010-08-04

**Authors:** Jiong-Peng Zhao, Fu-Chen Liu

**Affiliations:** a School of Chemistry and Chemical Engineering, Tianjin University of Technology, Tianjin 300191, People’s Republic of China

## Abstract

In the title complex, [Fe(C_2_O_4_)(C_5_H_4_N_2_O_4_)]_*n*_, the Fe^II^ ion is coordinated by two oxalate anions and a pyrimidine-2-carb­oxy­lic acid ligand in a slightly distorted octa­hedral geometry. Each oxalate anion chelates to two Fe^II^ ions, forming chains along the *a* axis. The chains are further connected by O—H⋯O and C—H⋯O hydrogen bonds, stabilizing the structure. An intra­molecular O—H⋯N inter­action results in a five-membered ring.

## Related literature

For related structures, see: Zhang *et al.* (2008 ▶).
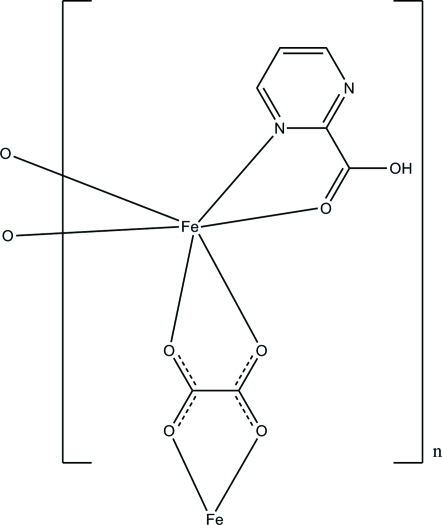


## Experimental

### 

#### Crystal data

[Fe(C_2_O_4_)(C_5_H_4_N_2_O_4_)]
*M* *_r_* = 267.97
Orthorhombic, 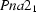

*a* = 9.0524 (18) Å
*b* = 9.1578 (18) Å
*c* = 11.329 (2) Å
*V* = 939.2 (3) Å^3^
*Z* = 4
Mo *K*α radiation
μ = 1.62 mm^−1^
*T* = 293 K
0.20 × 0.18 × 0.16 mm

#### Data collection

Rigaku SCXmini diffractometer
Absorption correction: multi-scan (*ABSCOR*; Higashi, 1995 ▶) *T*_min_ = 0.780, *T*_max_ = 1
7603 measured reflections
1658 independent reflections
1503 reflections with *I* > 2σ(*I*)
*R*_int_ = 0.074

#### Refinement

*R*[*F* ^2^ > 2σ(*F* ^2^)] = 0.040
*wR*(*F* ^2^) = 0.083
*S* = 1.11
1658 reflections
145 parameters
1 restraint
H-atom parameters constrained
Δρ_max_ = 0.46 e Å^−3^
Δρ_min_ = −0.30 e Å^−3^
Absolute structure: Flack (1983 ▶), 783 Friedel pairs
Flack parameter: 0.05 (3)

### 

Data collection: *SCXmini Benchtop Crystallography System Software* (Rigaku, 2006 ▶); cell refinement: *PROCESS-AUTO* (Rigaku, 1998 ▶); data reduction: *PROCESS-AUTO*; program(s) used to solve structure: *SHELXS97* (Sheldrick, 2008 ▶); program(s) used to refine structure: *SHELXL97* (Sheldrick, 2008 ▶); molecular graphics: *ORTEPIII* (Burnett & Johnson, 1996 ▶) and *PLATON* (Spek, 2009 ▶); software used to prepare material for publication: *SHELXTL* (Sheldrick, 2008 ▶).

## Supplementary Material

Crystal structure: contains datablocks global, I. DOI: 10.1107/S1600536810030023/pv2297sup1.cif

Structure factors: contains datablocks I. DOI: 10.1107/S1600536810030023/pv2297Isup2.hkl

Additional supplementary materials:  crystallographic information; 3D view; checkCIF report

## Figures and Tables

**Table 1:** Hydrogen-bond geometry (Å, °)

*D*—H⋯*A*	*D*—H	H⋯*A*	*D*⋯*A*	*D*—H⋯*A*
C6—H6*A*⋯O3^i^	0.93	2.48	3.279 (6)	145
O6—H6⋯O1^ii^	0.85	2.17	2.988 (5)	161
O6—H6⋯N2	0.85	2.40	2.743 (6)	105

Symmetry codes: (i) 
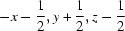
; (ii) 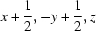.

**Fig. 1.**
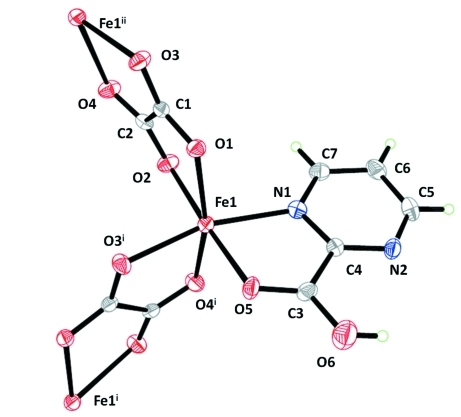
The coordinated mode and linkage of the complex. Ellipsoids are drawn at the 30% probability level. Symmetry codes: i = x+1/2,-y-1/2,z ii= x-1/2,-y-1/2,z

**Fig. 2.**
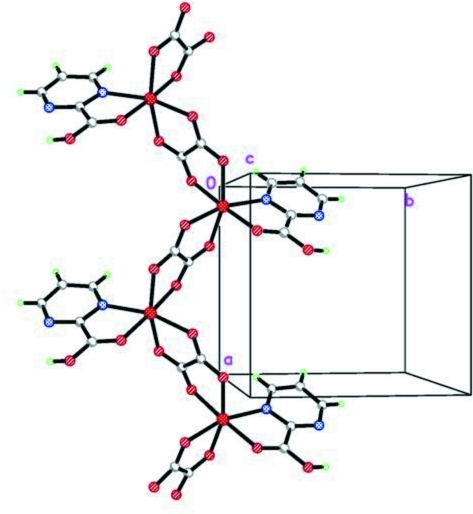
A partial packing diagram of the unit cell viewed down the c-axis.

## supplementary crystallographic information

### Comment

Pyrimidine-2-carboxylic acid (HL) and oxalate anion have similar coordination mode, acting as bidentate ligands, and some Cd complexes have been reported containing both ligands (Zhang *et al.*, 2008). Here we report the synthesis and crystal structure of a new iron complex with pyrimidine-2-carboxylic acid and oxalate as co-ligands.

In the title complex, the Fe^II^ ions are coordinated by one HL ligand and two oxalate anions in a slightly distorted octahedral geometry (Fig. 1). Each oxalate anion chelates to two Fe^II^ ions resulting a chain along the *a*-axis. There is an intramolecular interaction O6—H6···N2 resulting in a five membererd ring. The O—H···O and C—H···O type hydrogen bonds between the oxalate and HL ligands impart stability to the structure (Fig. 2).

### Experimental

A mixture of Fe(III) chloride (2 mmol), oxalate acid (2 mmol)and pyrimidine-2-carbonitrile (1 mmol), in 10 ml dimethyl formamate (DMF) solvent was sealed in a Teflon-lined stainless-steel Parr bomb was heated at 413 K for 48 h. Red crystals of the title complex were collected after the bomb was allowed to cool to room temperature; yield 20%.

### Refinement

The absolute structure of the title complex was determined by the Flack (1983) method. Hydrogen atoms were included in calculated positions and treated as riding on their parent atoms with O—H and C—H = 0.85 and 0.93 Å, respectively and *U*_iso_(H) = 1.2*U*_eq_(O/C).

### Crystal data

**Table:**

[Fe(C_2_O_4_)(C_5_H_4_N_2_O_4_)]	*F*(000) = 536
*M**_r_* = 267.97	*D*_x_ = 1.895 Mg m^−^^3^
Orthorhombic, *P**n**a*2_1_	Mo *K*α radiation, λ = 0.71073 Å
Hall symbol: P 2c -2n	Cell parameters from 8691 reflections
*a* = 9.0524 (18) Å	θ = 3.2–27.5°
*b* = 9.1578 (18) Å	µ = 1.62 mm^−^^1^
*c* = 11.329 (2) Å	*T* = 293 K
*V* = 939.2 (3) Å^3^	Block, red
*Z* = 4	0.2 × 0.18 × 0.16 mm

### Data collection

**Table:**

Rigaku SCXmini diffractometer	1658 independent reflections
Radiation source: fine-focus sealed tube	1503 reflections with *I* > 2σ(*I*)
graphite	*R*_int_ = 0.074
ω scans	θ_max_ = 25.0°, θ_min_ = 3.2°
Absorption correction: multi-scan (*ABSCOR*; Higashi, 1995)	*h* = −10→10
*T*_min_ = 0.780, *T*_max_ = 1	*k* = −10→10
7603 measured reflections	*l* = −13→13

### Refinement

**Table:**

Refinement on *F*^2^	Secondary atom site location: difference Fourier map
Least-squares matrix: full	Hydrogen site location: inferred from neighbouring sites
*R*[*F*^2^ > 2σ(*F*^2^)] = 0.040	H-atom parameters constrained
*wR*(*F*^2^) = 0.083	*w* = 1/[σ^2^(*F*_o_^2^) + (0.0339*P*)^2^ + 0.7762*P*] where *P* = (*F*_o_^2^ + 2*F*_c_^2^)/3
*S* = 1.11	(Δ/σ)_max_ = 0.001
1658 reflections	Δρ_max_ = 0.46 e Å^−^^3^
145 parameters	Δρ_min_ = −0.30 e Å^−^^3^
1 restraint	Absolute structure: Flack (1983), 783 Friedel pairs
Primary atom site location: structure-invariant direct methods	Flack parameter: 0.05 (3)

### Special details

**Table:**

Geometry. All esds (except the esd in the dihedral angle between two l.s. planes) are estimated using the full covariance matrix. The cell esds are taken into account individually in the estimation of esds in distances, angles and torsion angles; correlations between esds in cell parameters are only used when they are defined by crystal symmetry. An approximate (isotropic) treatment of cell esds is used for estimating esds involving l.s. planes.
Refinement. Refinement of *F*^2^ against ALL reflections. The weighted *R*-factor *wR* and goodness of fit *S* are based on *F*^2^, conventional *R*-factors *R* are based on *F*, with *F* set to zero for negative *F*^2^. The threshold expression of *F*^2^ > σ(*F*^2^) is used only for calculating *R*-factors(gt) *etc*. and is not relevant to the choice of reflections for refinement. *R*-factors based on *F*^2^ are statistically about twice as large as those based on *F*, and *R*- factors based on ALL data will be even larger.

### Fractional atomic coordinates and isotropic or equivalent isotropic displacement parameters (Å^2^)

**Table:**

	*x*	*y*	*z*	*U*_iso_*/*U*_eq_	
Fe1	0.13566 (6)	−0.08170 (6)	0.71653 (7)	0.02969 (18)	
C4	0.1749 (5)	0.2409 (5)	0.7015 (4)	0.0263 (10)	
O5	0.2520 (4)	0.0586 (3)	0.8376 (3)	0.0397 (9)	
O1	−0.0702 (3)	−0.0924 (3)	0.8009 (3)	0.0293 (7)	
N1	0.0989 (4)	0.1349 (4)	0.6452 (4)	0.0307 (9)	
O6	0.3406 (5)	0.2878 (5)	0.8630 (4)	0.0624 (12)	
H6	0.3456	0.3746	0.8369	0.075*	
C3	0.2601 (5)	0.1886 (5)	0.8074 (4)	0.0325 (11)	
O3	−0.2731 (3)	−0.2329 (4)	0.8046 (3)	0.0353 (8)	
O2	0.0098 (4)	−0.2138 (4)	0.5963 (3)	0.0343 (8)	
C7	0.0149 (5)	0.1768 (6)	0.5565 (4)	0.0349 (12)	
H7A	−0.0405	0.1072	0.5163	0.042*	
O4	−0.1781 (4)	−0.3696 (4)	0.6089 (3)	0.0368 (9)	
C5	0.0923 (6)	0.4168 (6)	0.5831 (5)	0.0438 (13)	
H5A	0.0916	0.5141	0.5597	0.053*	
C6	0.0066 (6)	0.3211 (6)	0.5216 (5)	0.0408 (13)	
H6A	−0.0539	0.3511	0.4598	0.049*	
N2	0.1764 (5)	0.3806 (4)	0.6738 (4)	0.0350 (11)	
C2	−0.1034 (5)	−0.2649 (5)	0.6452 (4)	0.0263 (11)	
C1	−0.1539 (5)	−0.1906 (5)	0.7602 (4)	0.0256 (10)	

### Atomic displacement parameters (Å^2^)

**Table:**

	*U*^11^	*U*^22^	*U*^33^	*U*^12^	*U*^13^	*U*^23^
Fe1	0.0248 (3)	0.0246 (3)	0.0396 (4)	−0.0008 (3)	0.0015 (4)	0.0021 (4)
C4	0.028 (2)	0.028 (2)	0.023 (3)	0.0011 (17)	0.002 (2)	−0.002 (2)
O5	0.053 (2)	0.0245 (19)	0.042 (2)	−0.0094 (17)	−0.0203 (18)	0.0093 (15)
O1	0.0252 (17)	0.0282 (17)	0.0344 (19)	0.0003 (15)	0.0030 (15)	−0.0040 (15)
N1	0.030 (2)	0.034 (2)	0.029 (2)	0.0011 (18)	0.0004 (18)	0.0003 (18)
O6	0.076 (3)	0.049 (3)	0.062 (3)	−0.010 (2)	−0.010 (2)	0.007 (2)
C3	0.033 (3)	0.035 (3)	0.029 (3)	−0.007 (2)	−0.006 (2)	−0.001 (2)
O3	0.0304 (19)	0.0338 (19)	0.042 (2)	−0.0084 (16)	0.0102 (17)	−0.0078 (17)
O2	0.0298 (18)	0.0426 (19)	0.0304 (18)	−0.0060 (16)	0.0071 (16)	−0.0054 (17)
C7	0.033 (3)	0.042 (3)	0.030 (3)	−0.002 (2)	−0.007 (2)	−0.003 (2)
O4	0.0292 (18)	0.041 (2)	0.040 (2)	−0.0042 (16)	0.0069 (17)	−0.0142 (17)
C5	0.053 (3)	0.033 (3)	0.045 (3)	0.010 (3)	−0.001 (3)	0.008 (3)
C6	0.043 (3)	0.043 (3)	0.037 (3)	0.010 (3)	−0.008 (3)	0.002 (2)
N2	0.041 (3)	0.024 (2)	0.039 (3)	−0.0041 (17)	−0.0069 (19)	0.0036 (16)
C2	0.023 (2)	0.030 (3)	0.026 (3)	0.007 (2)	−0.001 (2)	−0.001 (2)
C1	0.021 (2)	0.024 (2)	0.032 (2)	0.003 (2)	0.0027 (19)	0.0036 (18)

### Geometric parameters (Å, °)

**Table:**

Fe1—O1	2.097 (3)	O6—H6	0.8500
Fe1—O4^i^	2.128 (3)	O3—C1	1.252 (5)
Fe1—O3^i^	2.136 (3)	O3—Fe1^ii^	2.136 (3)
Fe1—O2	2.148 (3)	O2—C2	1.255 (6)
Fe1—O5	2.154 (3)	C7—C6	1.382 (7)
Fe1—N1	2.168 (4)	C7—H7A	0.9300
C4—N2	1.318 (6)	O4—C2	1.243 (6)
C4—N1	1.350 (6)	O4—Fe1^ii^	2.128 (3)
C4—C3	1.504 (7)	C5—N2	1.321 (7)
O5—C3	1.240 (6)	C5—C6	1.362 (8)
O1—C1	1.263 (6)	C5—H5A	0.9300
N1—C7	1.317 (6)	C6—H6A	0.9300
O6—C3	1.325 (6)	C2—C1	1.540 (6)
			
O1—Fe1—O4^i^	162.87 (14)	C3—O6—H6	120.4
O1—Fe1—O3^i^	95.38 (13)	O5—C3—O6	124.0 (4)
O4^i^—Fe1—O3^i^	78.16 (13)	O5—C3—C4	119.7 (4)
O1—Fe1—O2	77.96 (12)	O6—C3—C4	116.3 (4)
O4^i^—Fe1—O2	86.49 (14)	C1—O3—Fe1^ii^	113.1 (3)
O3^i^—Fe1—O2	93.08 (14)	C2—O2—Fe1	111.3 (3)
O1—Fe1—O5	99.90 (14)	N1—C7—C6	121.9 (5)
O4^i^—Fe1—O5	95.90 (15)	N1—C7—H7A	119.1
O3^i^—Fe1—O5	89.29 (13)	C6—C7—H7A	119.1
O2—Fe1—O5	176.94 (13)	C2—O4—Fe1^ii^	113.7 (3)
O1—Fe1—N1	94.37 (14)	N2—C5—C6	124.4 (5)
O4^i^—Fe1—N1	95.72 (14)	N2—C5—H5A	117.8
O3^i^—Fe1—N1	163.96 (15)	C6—C5—H5A	117.8
O2—Fe1—N1	101.40 (14)	C5—C6—C7	115.9 (5)
O5—Fe1—N1	76.50 (14)	C5—C6—H6A	122.0
N2—C4—N1	126.2 (5)	C7—C6—H6A	122.0
N2—C4—C3	119.6 (4)	C4—N2—C5	115.0 (4)
N1—C4—C3	114.2 (4)	O4—C2—O2	125.8 (5)
C3—O5—Fe1	115.2 (3)	O4—C2—C1	117.4 (4)
C1—O1—Fe1	113.6 (3)	O2—C2—C1	116.8 (4)
C7—N1—C4	116.5 (4)	O3—C1—O1	126.2 (4)
C7—N1—Fe1	129.7 (4)	O3—C1—C2	117.3 (4)
C4—N1—Fe1	113.8 (3)	O1—C1—C2	116.5 (4)
			
O1—Fe1—O5—C3	−99.7 (4)	N1—C4—C3—O5	−2.3 (6)
O4^i^—Fe1—O5—C3	86.9 (4)	N2—C4—C3—O6	−4.2 (7)
O3^i^—Fe1—O5—C3	165.0 (4)	N1—C4—C3—O6	177.7 (4)
N1—Fe1—O5—C3	−7.5 (4)	O1—Fe1—O2—C2	−17.7 (3)
O4^i^—Fe1—O1—C1	−10.5 (6)	O4^i^—Fe1—O2—C2	155.1 (3)
O3^i^—Fe1—O1—C1	−77.3 (3)	O3^i^—Fe1—O2—C2	77.2 (3)
O2—Fe1—O1—C1	14.7 (3)	N1—Fe1—O2—C2	−109.8 (3)
O5—Fe1—O1—C1	−167.5 (3)	C4—N1—C7—C6	1.0 (7)
N1—Fe1—O1—C1	115.5 (3)	Fe1—N1—C7—C6	−178.9 (4)
N2—C4—N1—C7	−2.3 (7)	N2—C5—C6—C7	−2.2 (9)
C3—C4—N1—C7	175.7 (4)	N1—C7—C6—C5	1.0 (8)
N2—C4—N1—Fe1	177.7 (4)	N1—C4—N2—C5	1.2 (7)
C3—C4—N1—Fe1	−4.3 (5)	C3—C4—N2—C5	−176.7 (4)
O1—Fe1—N1—C7	−74.8 (4)	C6—C5—N2—C4	1.2 (8)
O4^i^—Fe1—N1—C7	91.3 (4)	Fe1^ii^—O4—C2—O2	−178.8 (4)
O3^i^—Fe1—N1—C7	157.9 (5)	Fe1^ii^—O4—C2—C1	1.0 (5)
O2—Fe1—N1—C7	3.8 (4)	Fe1—O2—C2—O4	−162.4 (4)
O5—Fe1—N1—C7	−174.0 (4)	Fe1—O2—C2—C1	17.8 (5)
O1—Fe1—N1—C4	105.3 (3)	Fe1^ii^—O3—C1—O1	−173.3 (4)
O4^i^—Fe1—N1—C4	−88.6 (3)	Fe1^ii^—O3—C1—C2	6.6 (5)
O3^i^—Fe1—N1—C4	−22.1 (7)	Fe1—O1—C1—O3	169.6 (4)
O2—Fe1—N1—C4	−176.2 (3)	Fe1—O1—C1—C2	−10.3 (5)
O5—Fe1—N1—C4	6.1 (3)	O4—C2—C1—O3	−5.4 (6)
Fe1—O5—C3—O6	−172.2 (4)	O2—C2—C1—O3	174.4 (4)
Fe1—O5—C3—C4	7.8 (6)	O4—C2—C1—O1	174.4 (4)
N2—C4—C3—O5	175.8 (5)	O2—C2—C1—O1	−5.7 (6)

Symmetry codes: (i) *x*+1/2, −*y*−1/2, *z*; (ii) *x*−1/2, −*y*−1/2, *z*.

### Hydrogen-bond geometry (Å, °)

**Table:**

*D*—H···*A*	*D*—H	H···*A*	*D*···*A*	*D*—H···*A*
C6—H6A···O3^iii^	0.93	2.48	3.279 (6)	145
O6—H6···O1^iv^	0.85	2.17	2.988 (5)	161
O6—H6···N2	0.85	2.40	2.743 (6)	105

Symmetry codes: (iii) −*x*−1/2, *y*+1/2, *z*−1/2; (iv) *x*+1/2, −*y*+1/2, *z*.
